# Supplementary material for: Chamigrane-Type Sesquiterpenes from Laurencia dendroidea as Lead Compounds against Naegleria fowleri
Source: Mar Drugs. 2023 Mar 31;21(4):224. doi: 10.3390/md21040224 (PMC10143968; doi:10.3390/md21040224)
Supplement: Supplementary file 1 [file marinedrugs-21-00224-s001.zip › marinedrugs-2310588-supplementary.pdf]

Supplementary Material

Chamigrane-type sesquiterpenes from *Laurencia dendroidea* as lead compounds against *Naegleria fowleri*

Iñigo Arberas-Jiménez[1,2,a], Nathália Nocchi[3,4,a], Javier Chao-Pellicer[1,2,5], Ines Sifaoui[1,2], Angélica Ribeiro Soares[6], Ana R. Díaz-Marrero[3,7], José J. Fernández[3,4,*], José E. Piñero[1,2,5,*], Jacob Lorenzo-Morales[1,2,5,*]

[1] Instituto Universitario de Enfermedades Tropicales y Salud Pública de Canarias, Universidad de La Laguna (ULL), Avda. Astrofísico Fco. Sánchez, S/N, 38206 La Laguna, Tenerife, Islas Canarias, Spain

[2] Departamento de Obstetricia y Ginecología, Pediatría, Medicina Preventiva y Salud Pública, Toxicología, Medicina Legal y Forense y Parasitología, Universidad de La Laguna (ULL), Tenerife, Islas Canarias, Spain

[3] Instituto Universitario de Bio-Orgánica Antonio González (IUBO AG), Universidad de La Laguna (ULL), Avda. Astrofísico Fco. Sánchez 2, 38206 La Laguna, Tenerife, Islas Canarias, Spain.

[4] Departamento de Química Orgánica, Universidad de La Laguna (ULL), Avda. Astrofísico Fco. Sánchez 3, 38206 La Laguna, Tenerife, Spain.

[5] Consorcio Centro de Investigación Biomédica En Red (CIBER) de Enfermedades Infecciosas (CIBERINFEC), Instituto de Salud Carlos III, 28006 Madrid, Spain

[6] Universidade Federal do Rio de Janiero, Instituto de Biodiversidade e Sustentabilidade, Avda. São José do Barreto, 764, 27965-045 Macaé, RJ, Brazil

[7] Instituto de Productos Naturales y Agrobiología (IPNA), Consejo Superior de Investigaciones Científicas (CSIC), Avda. Astrofísico Fco. Sánchez 3, 38206 La Laguna, Tenerife, Islas Canarias, Spain

* Correspondence: jjfercas@ull.edu.es (J.J.F.); jpinero@ull.edu.es (J.E.P); jmlorenz@ull.edu.es (J.L.M.)

[a] I.A.J. and N.N. contributed equally to this work.

.

Table of Contents

CONTENTS	CAPTION	PAGE
**Figure S1**	^{1}H NMR spectrum of (+)-elatol (**1**) at 600 MHz in $CDCl_3$	S3
**Figure S2**	^{13}C NMR spectrum of (+)-elatol (**1**) at 600 MHz in $CDCl_3$	S4
**Figure S3**	COSY NMR spectrum of (+)-elatol (**1**) at 600 MHz in $CDCl_3$	S5
**Figure S4**	COSY NMR spectrum of (+)-elatol (**1**) at 600 MHz in $CDCl_3$	S6
**Figure S5**	HMBC NMR spectrum of (+)-elatol (**1**) at 600 MHz in $CDCl_3$	S7
**Figure S6**	ROESY spectrum of (+)-elatol (**1**) at 600 MHz in $CDCl_3$	S8
**Figure S7**	^{1}H NMR spectrum of (-)-elatol (**2**) at 600 MHz in $CDCl_3$	S9
**Figure S8**	^{13}C NMR spectrum of (-)-elatol (**2**) at 600 MHz in $CDCl_3$	S10
**Figure S9**	COSY NMR spectrum of (-)-elatol (**1**) at 600 MHz in $CDCl_3$	S11
**Figure S10**	HSQC NMR spectrum of (-)-elatol (**2**) at 600 MHz in $CDCl_3$	S12
**Figure S11**	HMBC NMR spectrum of (-)-elatol (**2**) at 600 MHz in $CDCl_3$	S13
**Figure S12**	ROESY spectrum of (-)-elatol (**2**) at 600 MHz in $CDCl_3$	S14
**Figure S13**	Higher magnification of Figure 2 (x100)	S15
**Figure S14**	Higher magnification of Figure 3 (x100)	S16
**Figure S15**	Higher magnification of Figure 4 (x100)	S17
**Figure S16**	Higher magnification of Figure 5 (x100)	S18

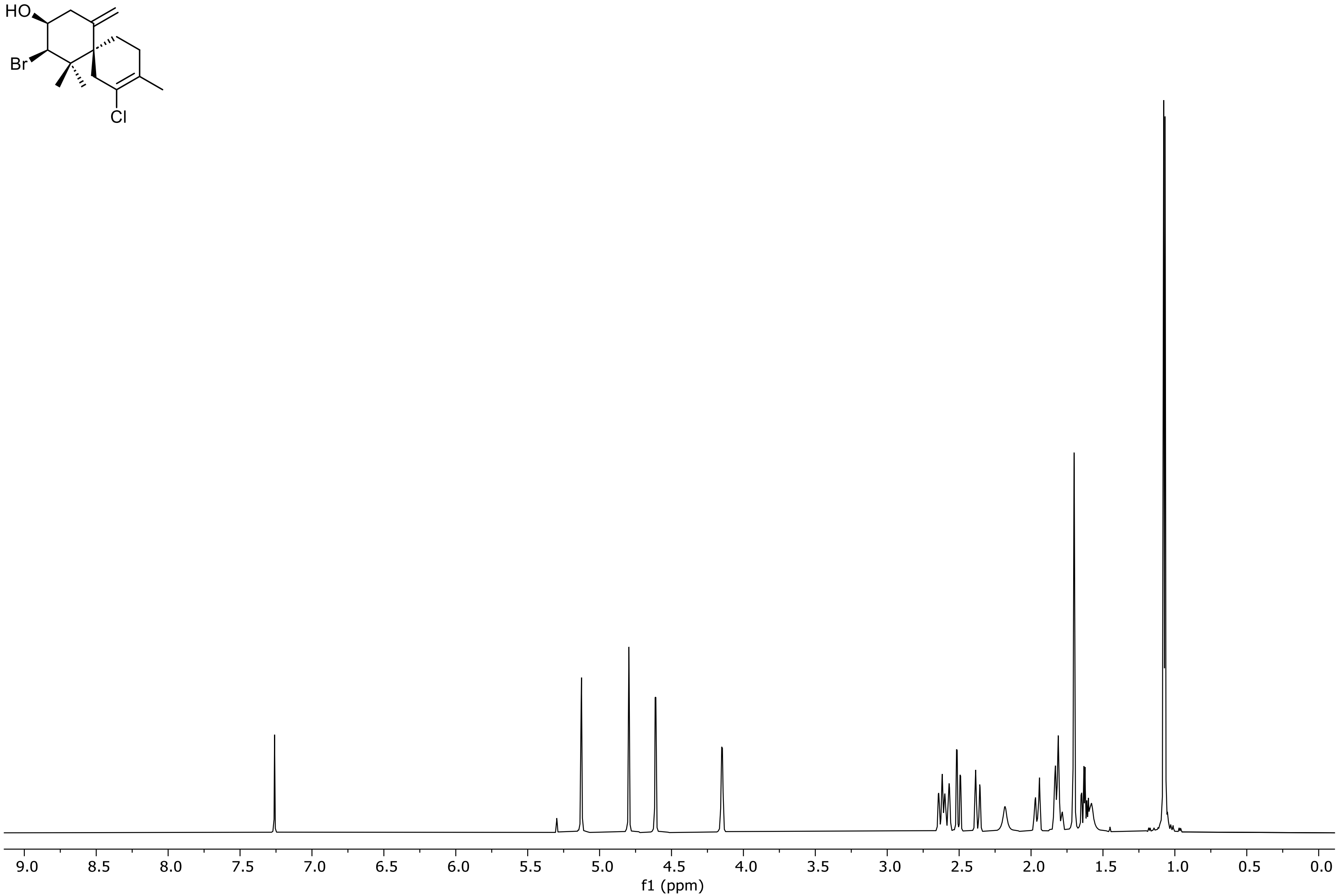

Figure S1: ^{1}H NMR spectrum of (+)-elatol (**1**) at 600 MHz in $CDCl_3$

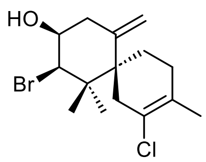

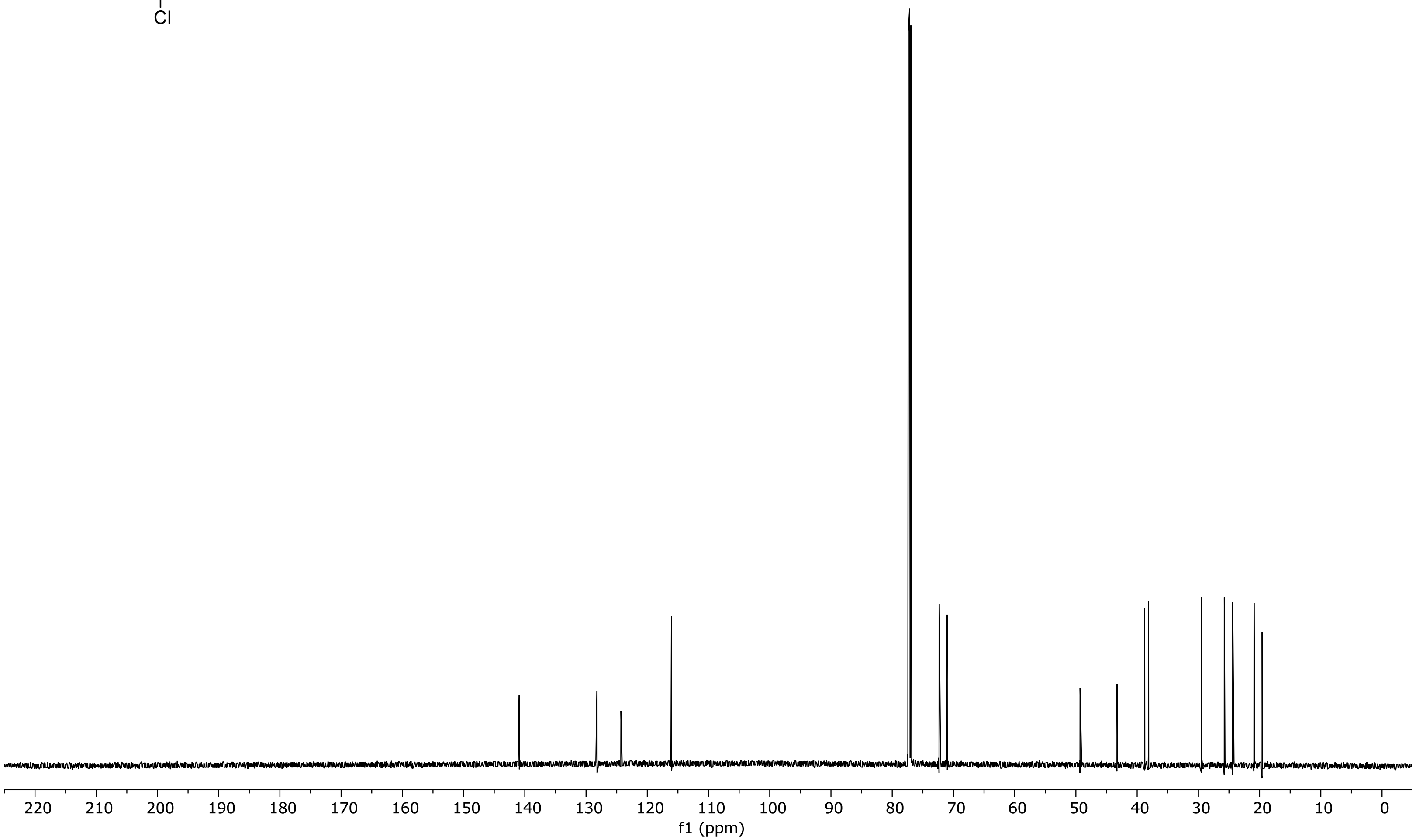

Figure S2: ^{13}C NMR spectrum of (+)-elatol (**1**) at 600 MHz in $CDCl_3$

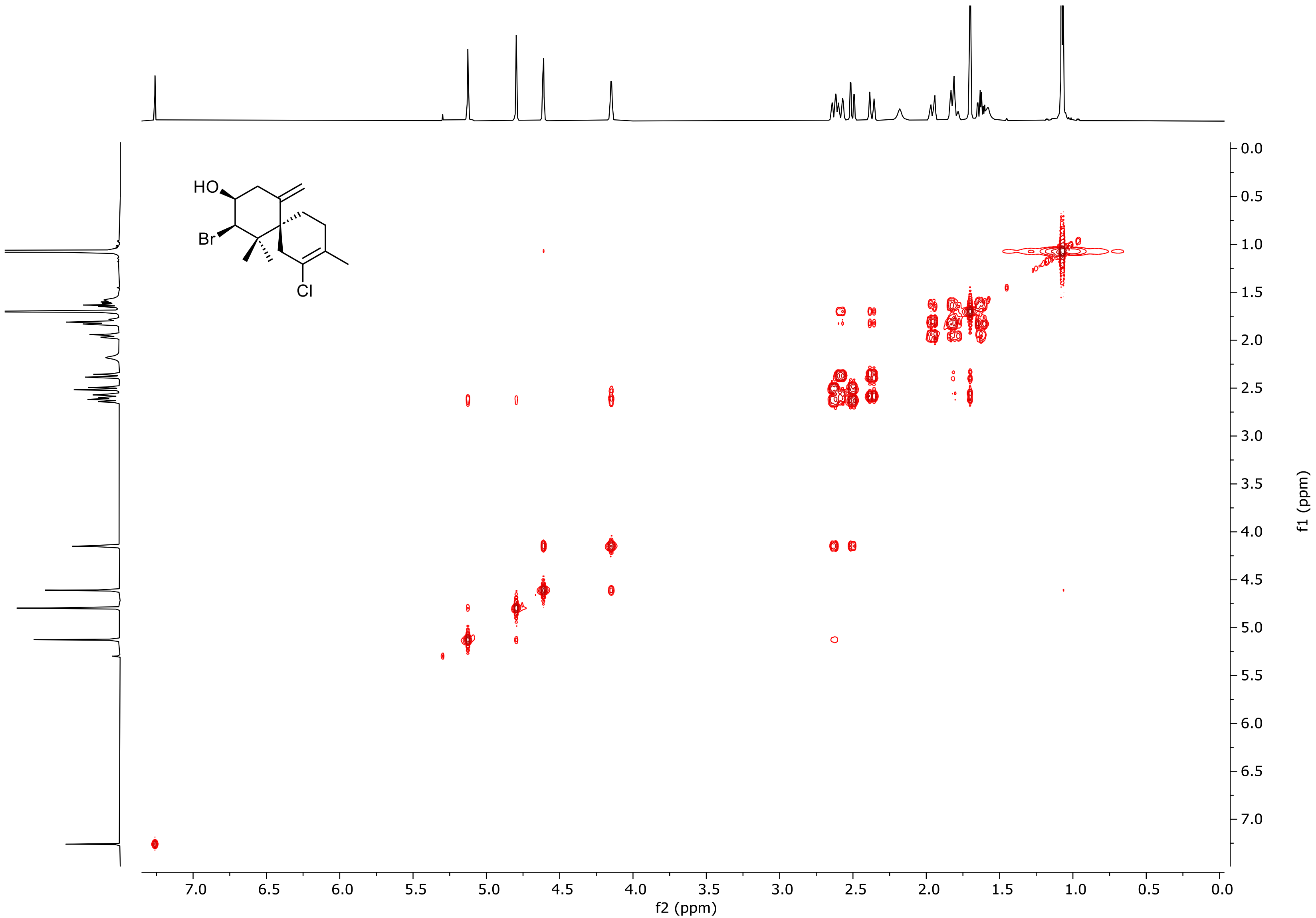

Figure S3: COSY NMR spectrum of (+)-elatol (**1**) at 600 MHz in $CDCl_3$

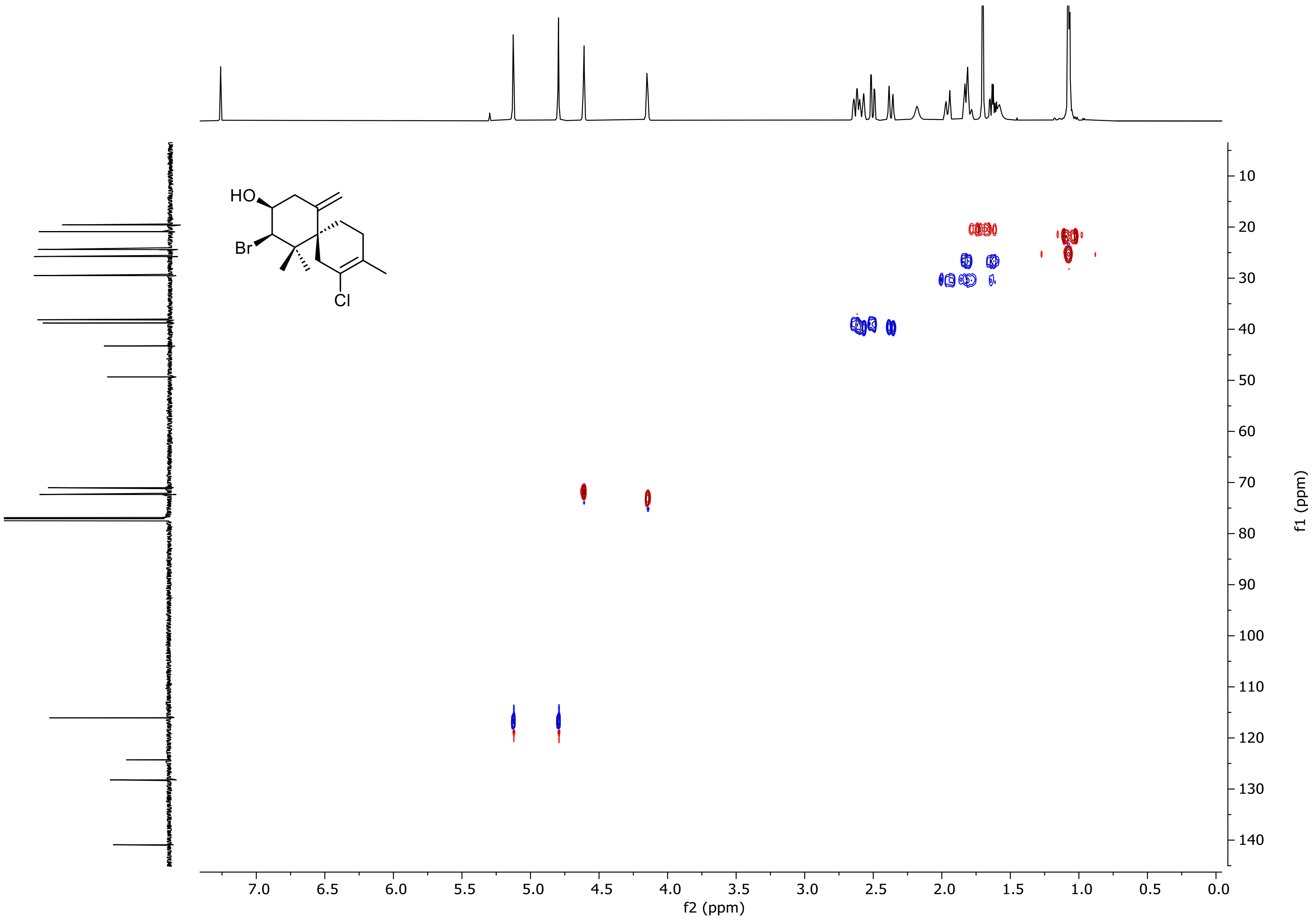

Figure S3: COSY NMR spectrum of (+)-elatol (**1**) at 600 MHz in $CDCl_3$

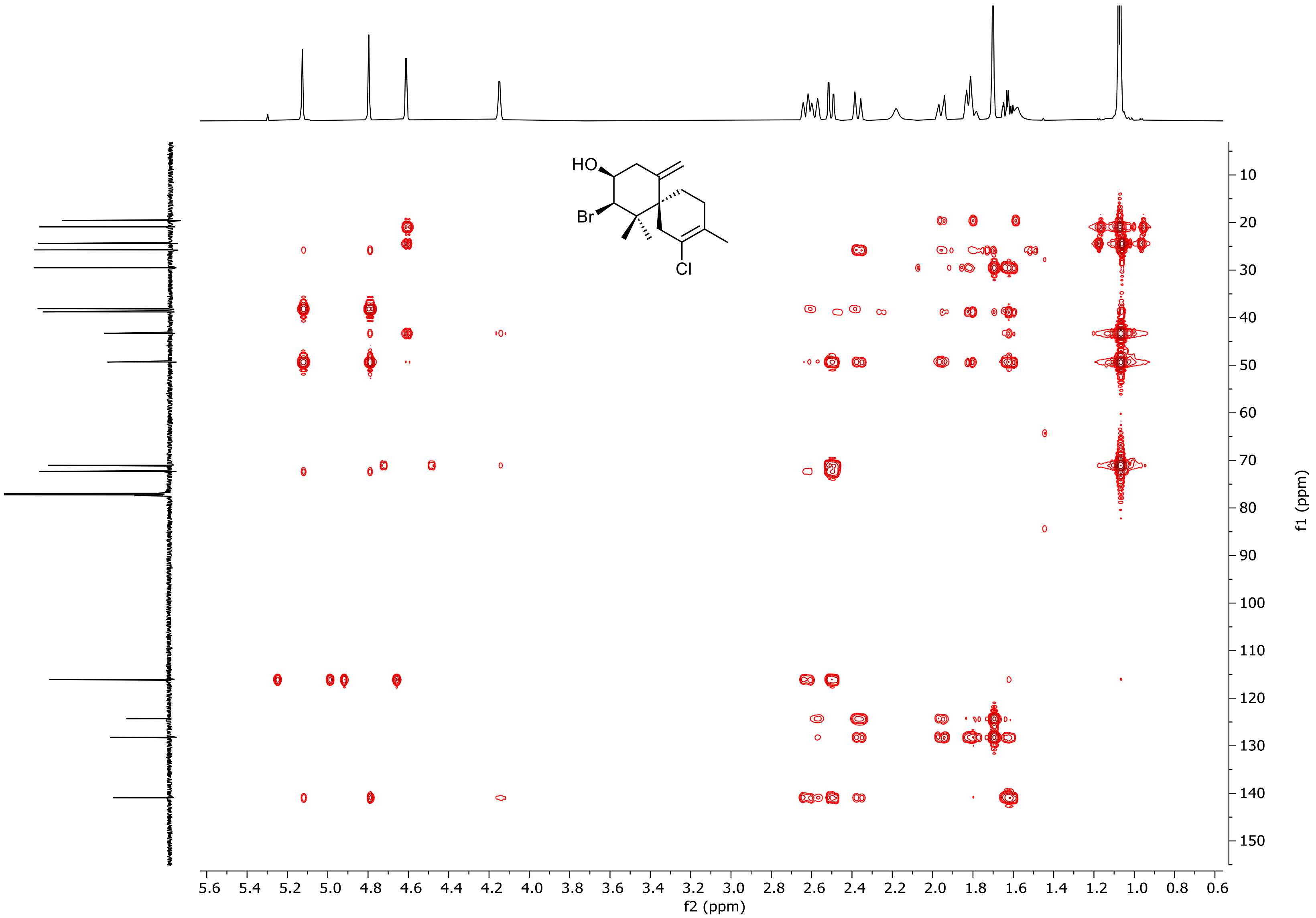

Figure S5: HMBC NMR spectrum of (+)-elatol (**1**) at 600 MHz in $CDCl_3$

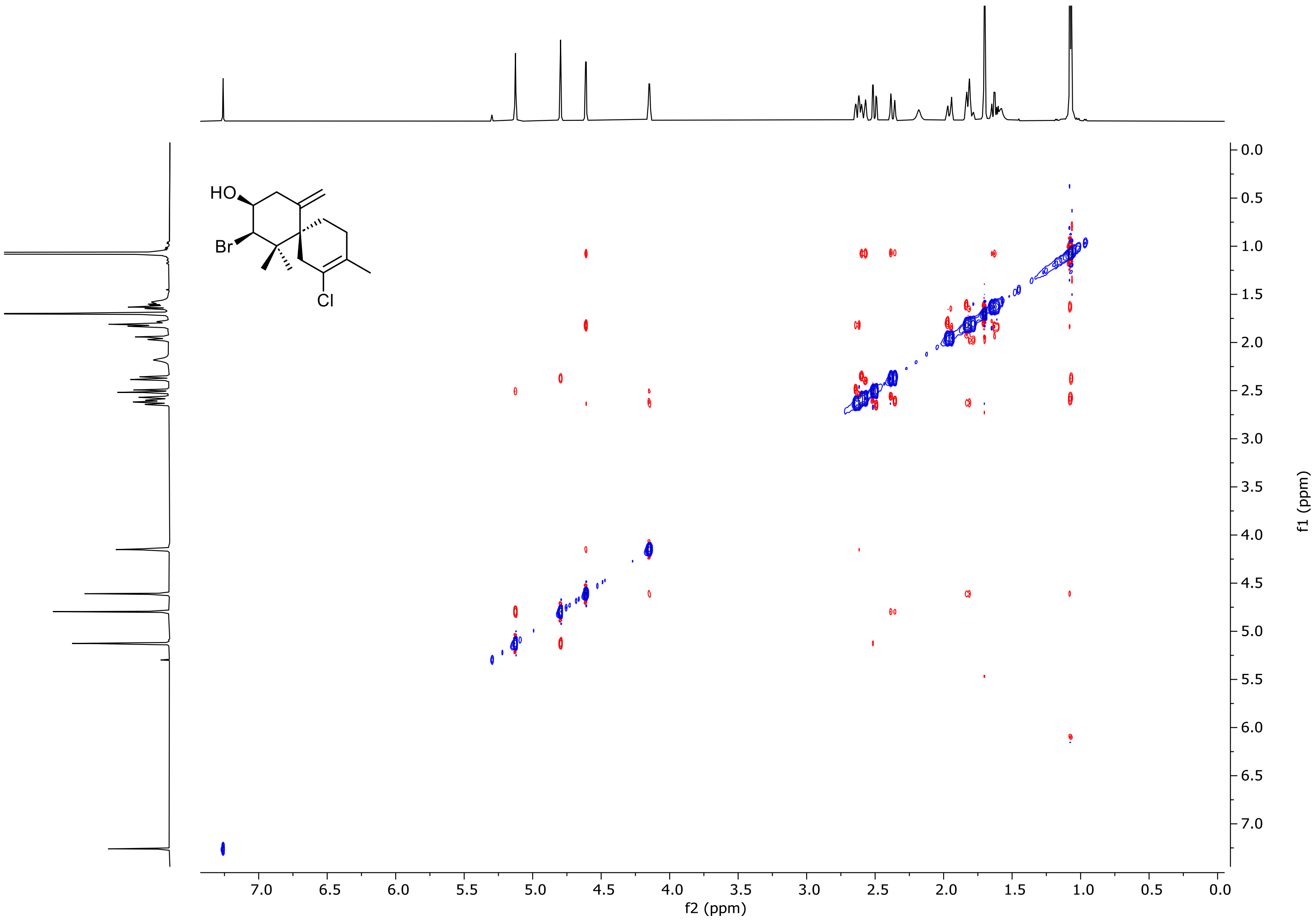

Figure S6: ROESY spectrum of (+)-elatol (**1**) at 600 MHz in $CDCl_3$

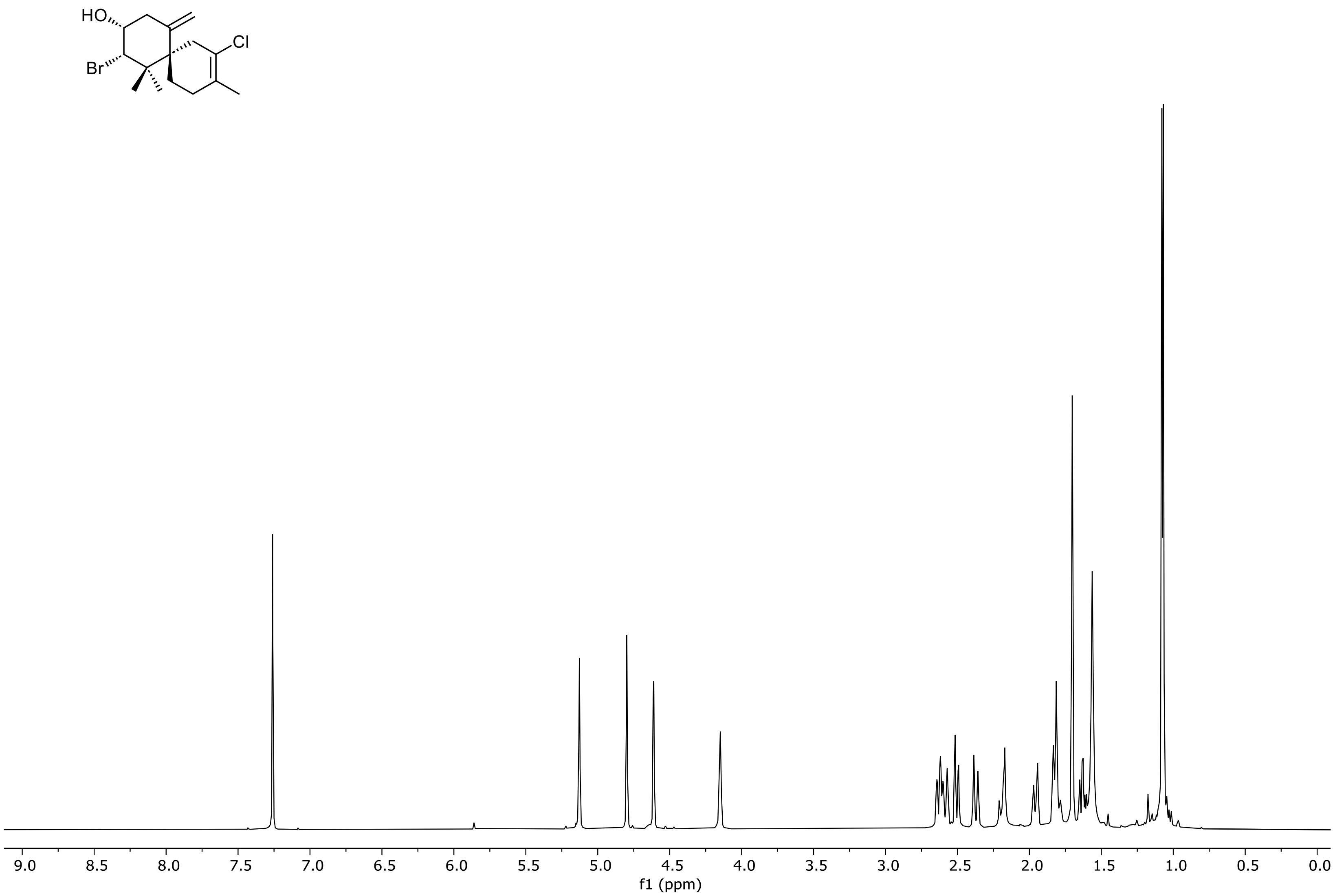

Figure S7: ^{1}H NMR spectrum of (-)-elatol (**2**) at 600 MHz in $CDCl_3$

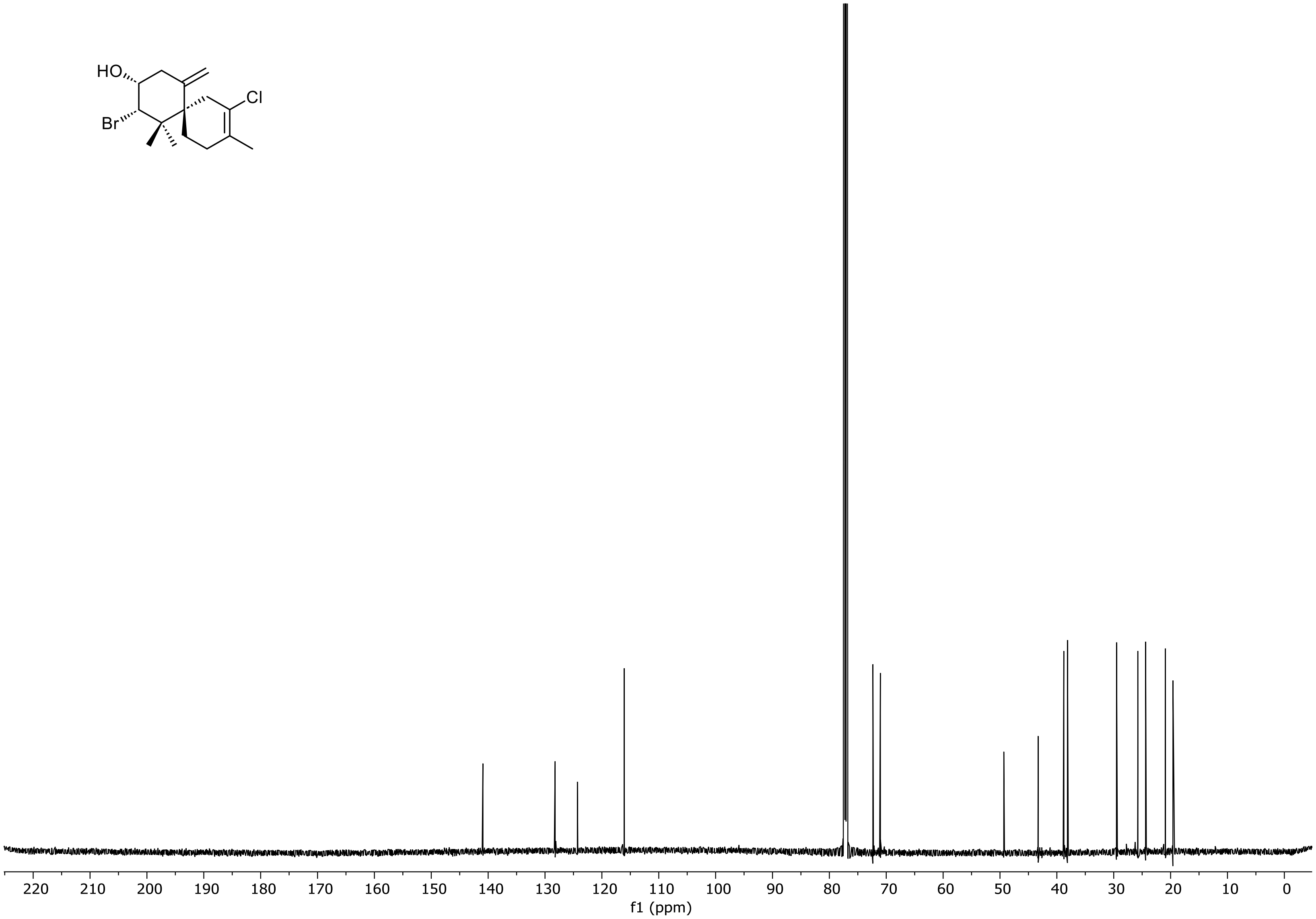

Figure S8: ^{13}C NMR spectrum of (-)-elatol (**2**) at 600 MHz in $CDCl_3$

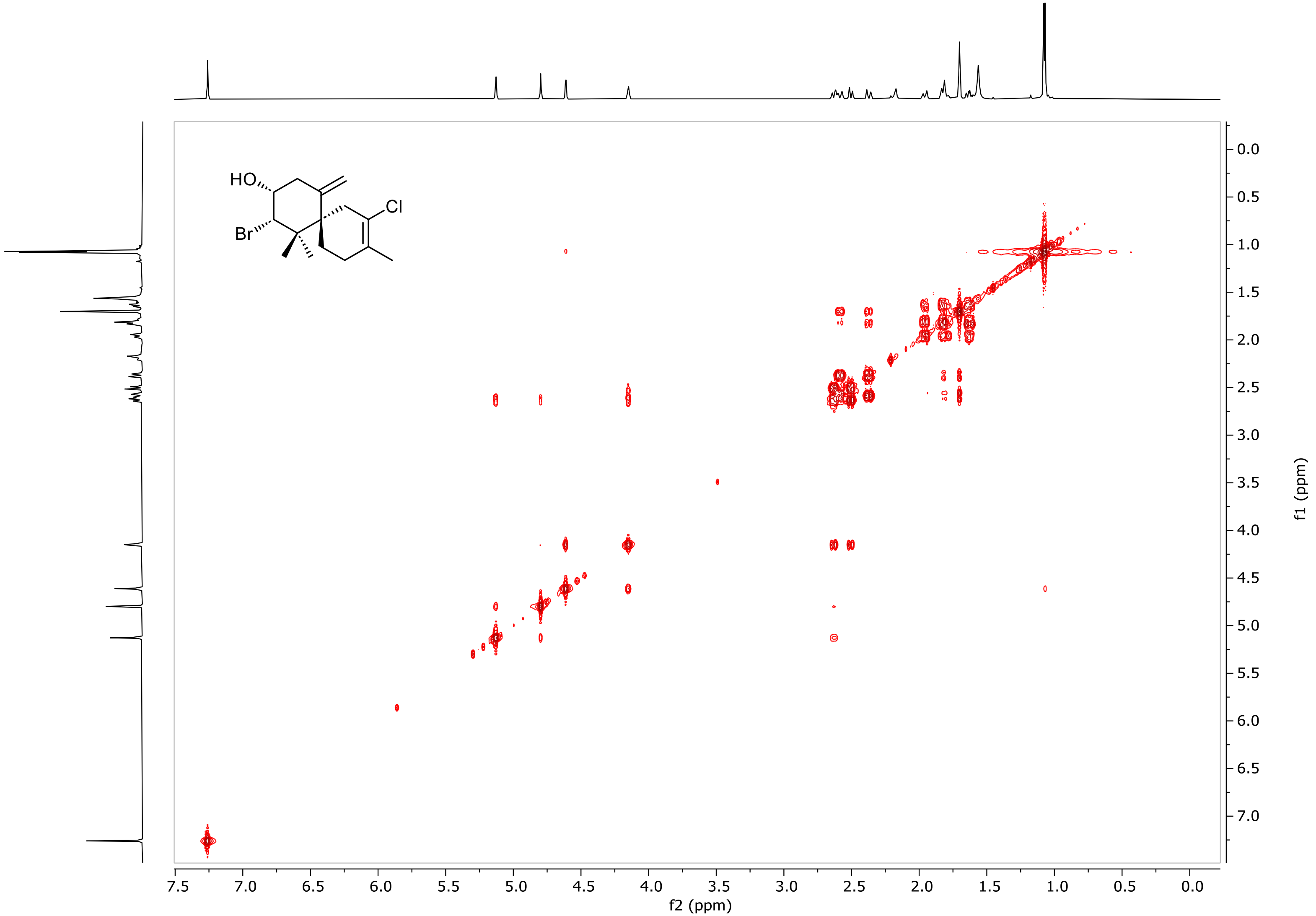

Figure S9: COSY NMR spectrum of (-)-elatol (**1**) at 600 MHz in $CDCl_3$

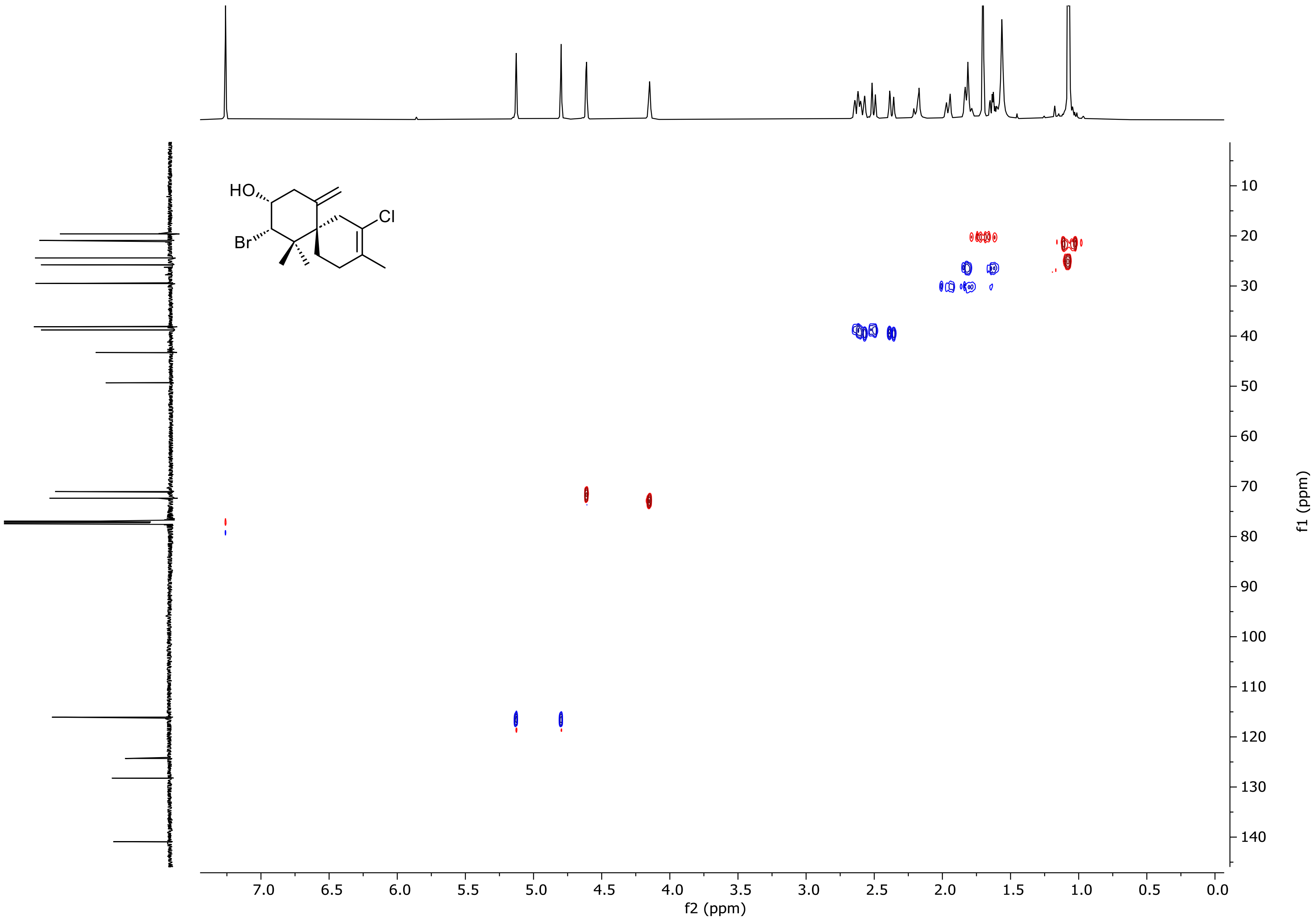

Figure S10: HSQC NMR spectrum of (-)-elatol (**2**) at 600 MHz in $CDCl_3$

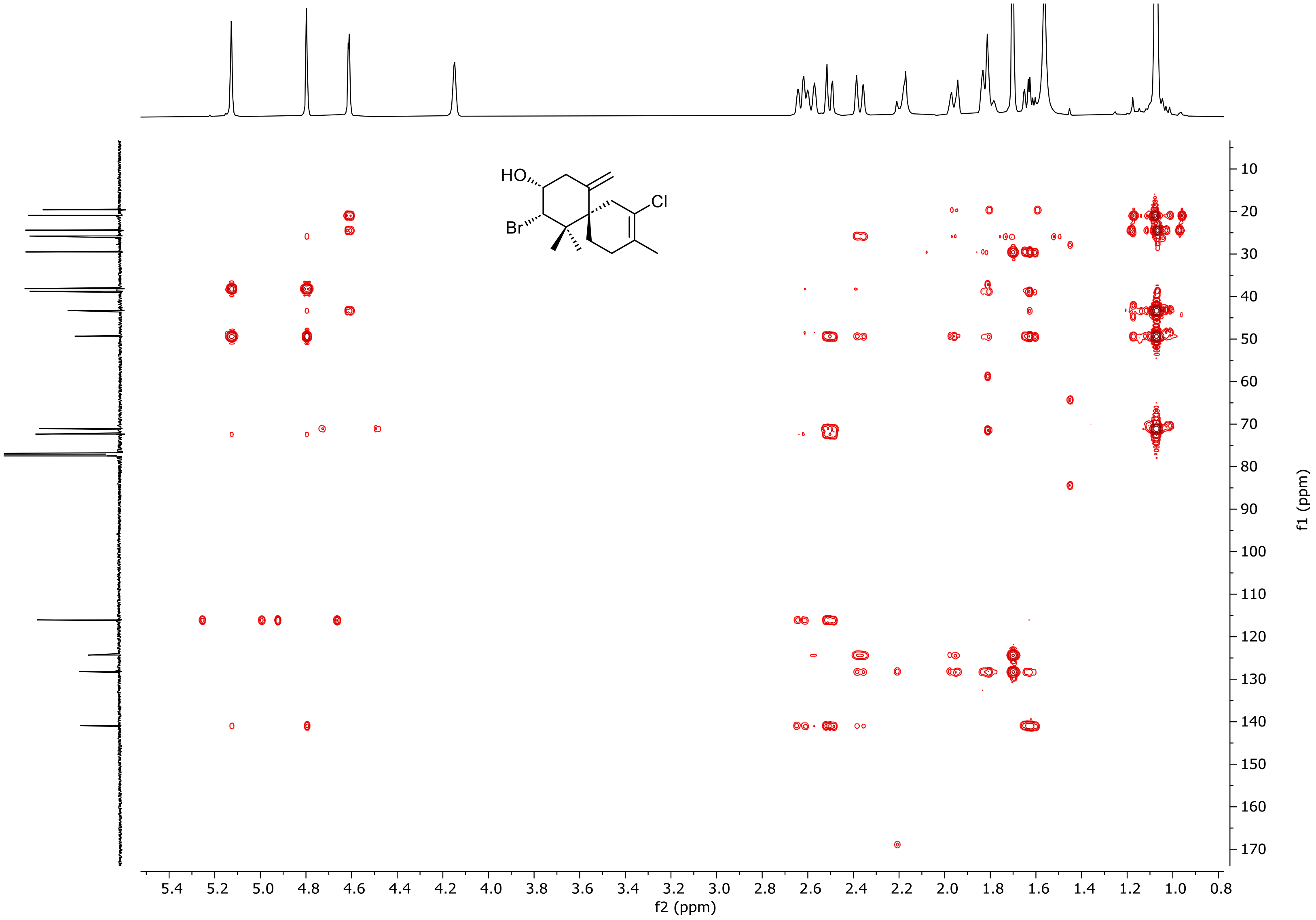

Figure S11: HMBC NMR spectrum of (-)-elatol (**2**) at 600 MHz in $CDCl_3$

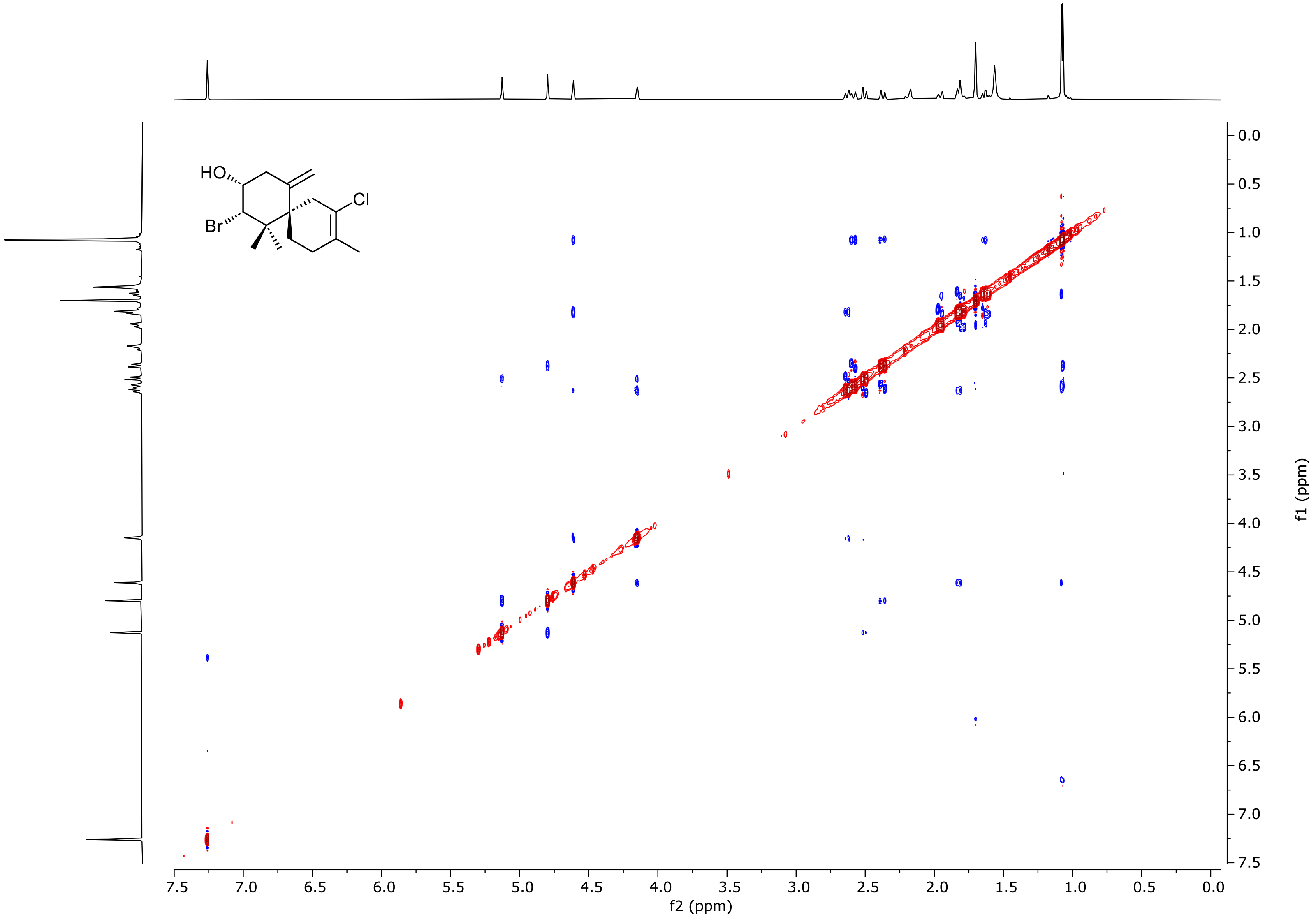

Figure S12: ROESY spectrum of (-)-elatol (**2**) at 600 MHz in $CDCl_3$

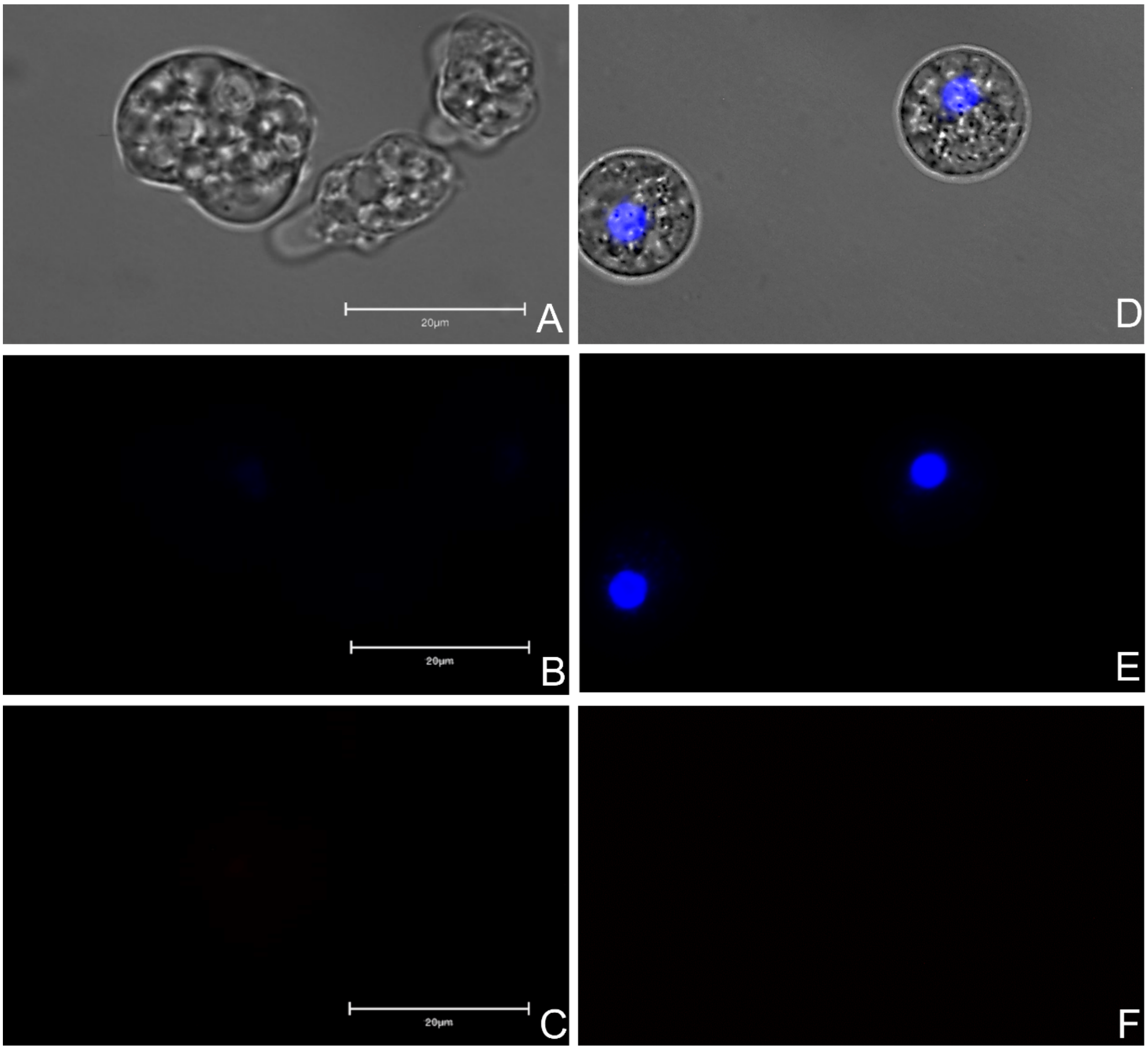

Figure S13. Higher magnification of Figure 2 (x100). (**D**-**F**) images show early apoptotic cells with intense blue fluorescence and no red fluorescence. Overlay channel (**A** and **D**); Hoechst channel (**B** and **E**), and propidium iodide channel (**C** and **F**). Images are representative of the cell population observed in the performed experiments. Images were obtained using an EVOS M500 Cell Imaging System, Life Technologies, Spain. (Scale bar: 20 µm)

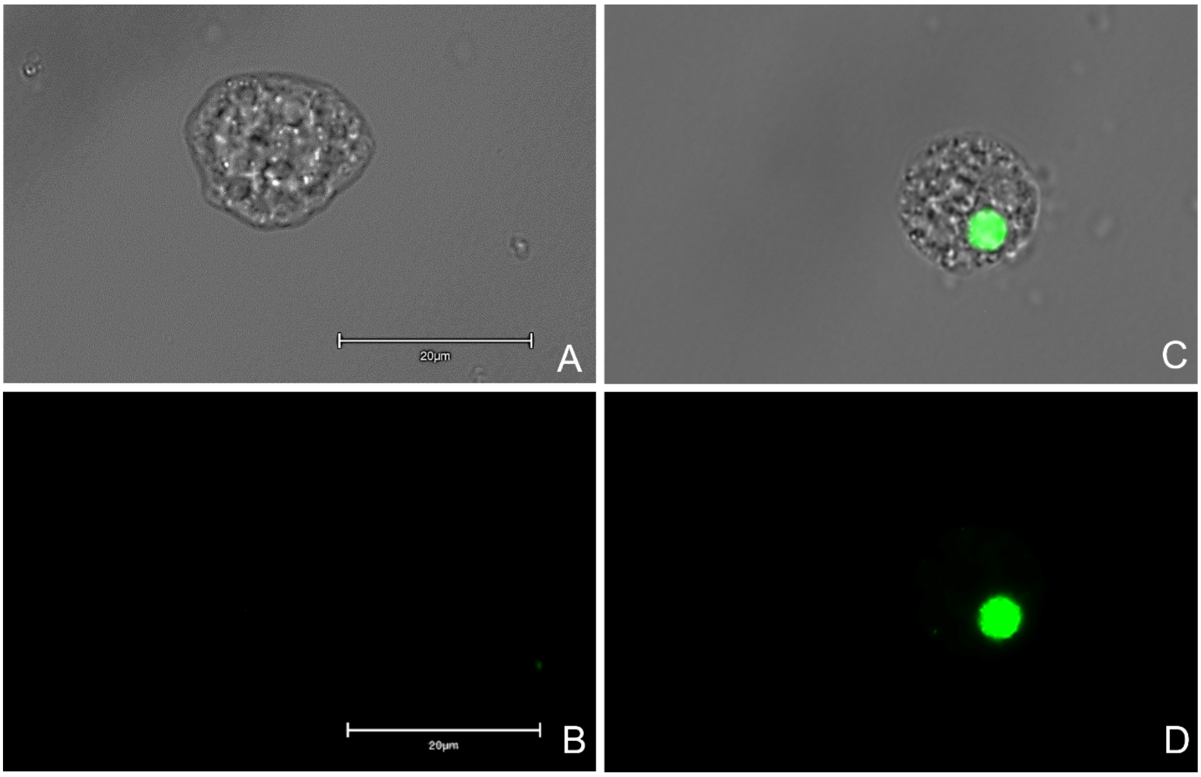

Figure S14. Higher magnification of Figure 3 (x100). Cells treated with the IC_{90} of (+)- Elatol showing green fluorescense(**C** and **D**), negative control (**A** and **B**). Overlay channel (**A** and **C**) and Sytox green channel (**B** and **D**). Images are representative of the cell population observed in the performed experiments. Images were obtained using an EVOS M500 Cell Imaging System, Life Technologies, Spain.

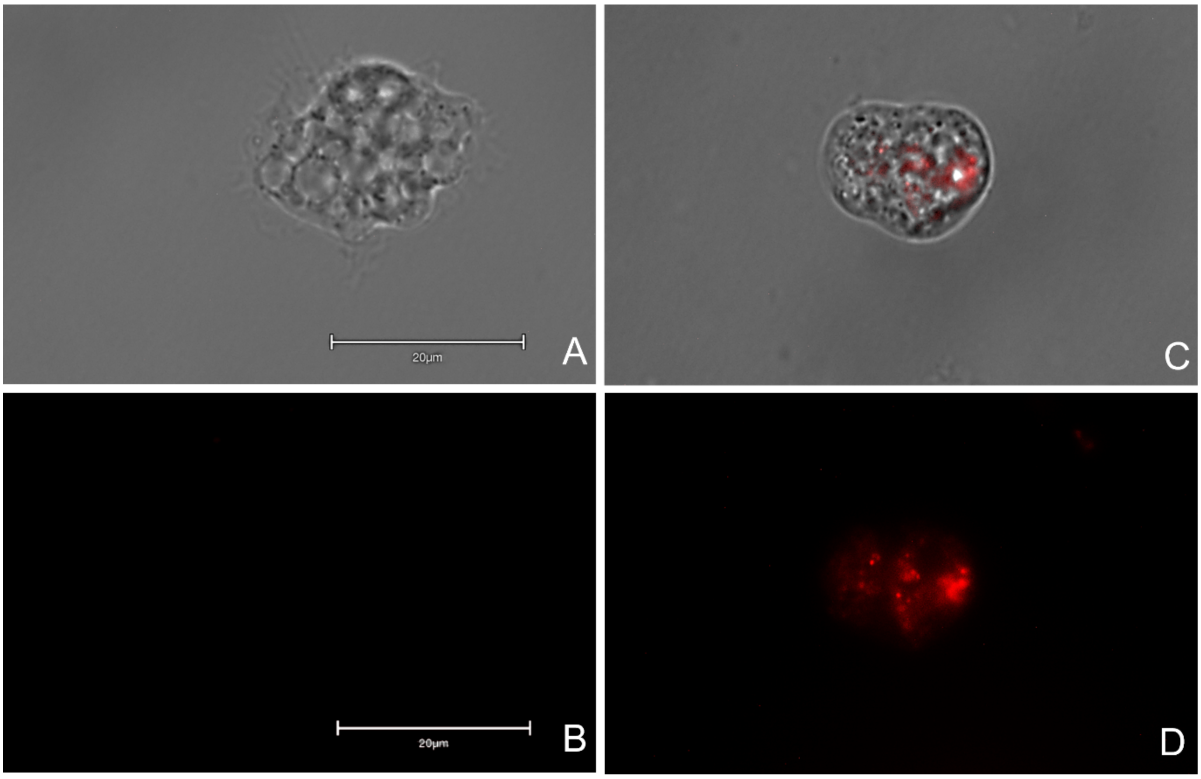

Figure S15. Higher magnification of Figure 4 (x100). Cells treated with the IC_{90} of (+)- Elatol showing red fluorescense (**C** and **D**), negative control (**A** and **B**). Overlay channel (**A** and **C**) and CellROX® channel (**B** and **D**). Images are representative of the cell population observed in the performed experiments. Images were obtained using an EVOS M500 Cell Imaging System, Life Technologies, Spain.

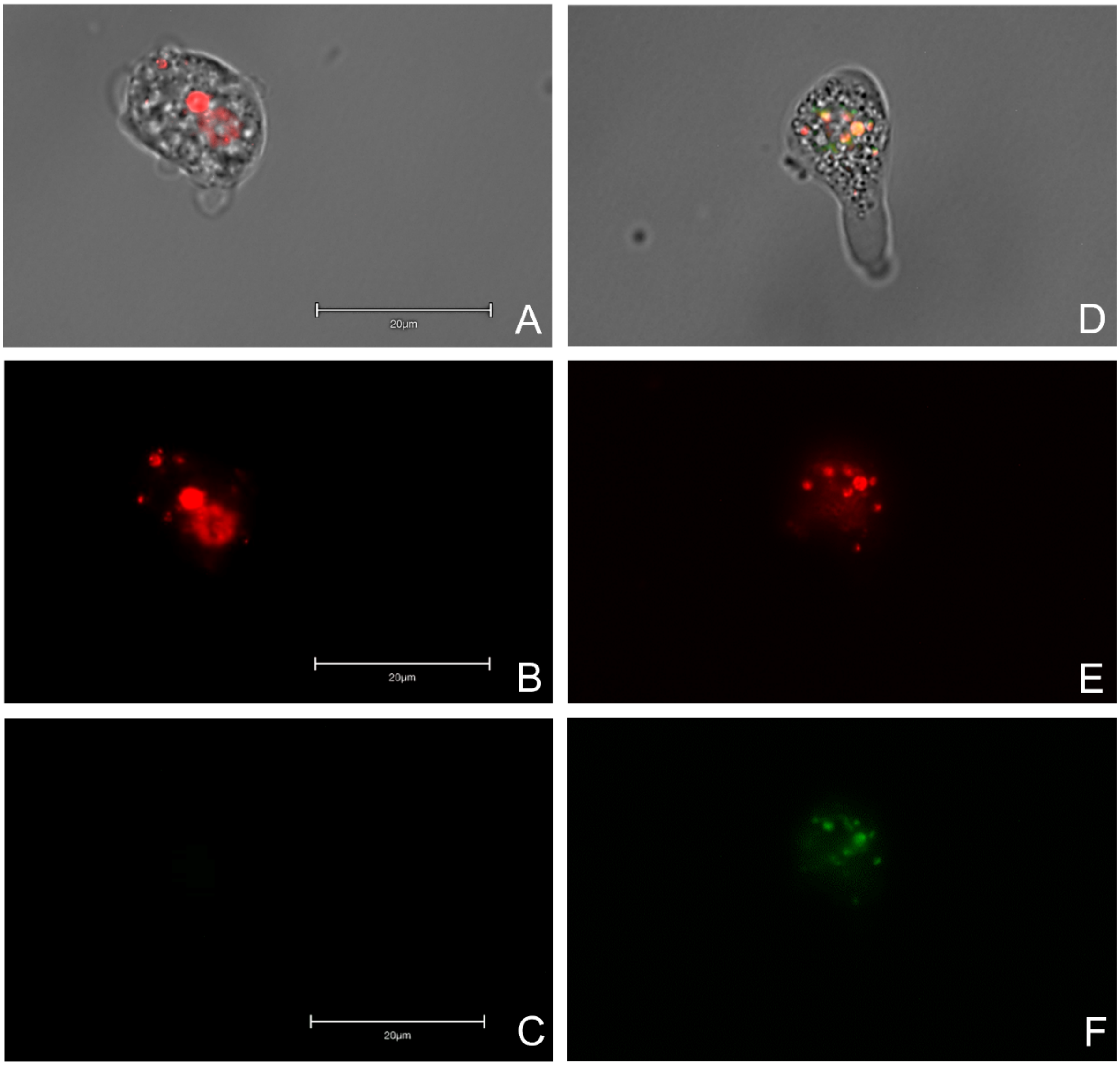

Figure S16. Higher magnification of Figure 5 (x100). Cells treated with the IC_{90} of (+)- Elatol showing red fluorescense (**C** and **D**), negative control (**A** and **B**). Overlay channel (**A** and **C**) and CellROX® channel (**B** and **D**). Images are representative of the cell population observed in the performed experiments. Images were obtained using an EVOS M500 Cell Imaging System, Life Technologies, Spain.
